# The Treatment of Insanity: Existing Defects and their Remedy

**Published:** 1907-05-11

**Authors:** 


### THE TREATMENT OF INSANITY.

### Existing Defects and their Remedy.

### V.—HOSPITAL AND ASYLUM METHODS CONTRASTED.

In real medical treatment, directed to the discovery of the causes, nature, and cure of insanity, very little has been done, very little is being done, and the methods found fertile in other fields are not being pursued. Why is this?

The causes are manifold. First and chief of all is the fact that when we compare the large general hospital with the large public asylum, we are comparing things which are in their nature different, and have no true basis of comparison. The general hospital is supported by endowment, or by voluntary contributions, or both. The public asylum is supported by the rates. It is administered by men who are in duty bound to incur no expense that is not strictly necessary for the humane care of their charges. If they desire to engage men in research work, they must pay them, and pay them well. It is easy to attract first-rate men to serve a great general hospital for little or nothing. They get their reward indirectly from their known connection with the institution. No one will serve an asylum on the same terms. The same collateral and indirect advantages are not to be had. On the contrary, situated as most asylums are, in remote country districts, a man who joins their service buries himself, and puts himself out of touch with opportunities and means of distinction. In the hospital, the care of the sick, the advancement of medical science, and the training of medical men in their profession, are co-ordinate aims, each of them properly pertaining to the institution, and each reinforcing and assisting the others. The lunatic asylum has no such triple aim. It is founded and maintained for the care of the insane only—that those whose mental disorder renders them obnoxious to the community may be cared for by the community at the least cost that is consistent with a large and enlightened humanity. They are comparable, not with the great general charitable hospitals, in which so much is done to advance the science and art of medicine, but with the great Poor-law infirmaries, in which no such function is recognised, no such aim followed, but in which the sick are cared for, at the expense of the rates, at the lowest cost compatible with efficiency.

When a young man takes a subordinate post in a general hospital—a dressership, or a clinical clerkship—he plunges into a keen intellectual atmosphere, and finds himself surrounded by other young fellows with whom he is in friendly competition and rivalry. His progress depends upon his professional attainments. The better he equips himself with medical and surgical knowledge and skill, the better are his chances of promotion to higher posts in the resident staff, and ultimately to that inclusion in the visiting staff which is the object of ambition to himself and all his fellows. With the young man who joins the staff of the public asylum it is far otherwise. His professional attainments, his knowledge of and skill in his profession will not advance his prospects in the least. The committee, to which he has to look for promotion, will not value these qualities in the least. They desire, and rightly desire, first of all the man who is an efficient administrator; who will manage well; who will preserve discipline and cultivate contentment in a large staff; who will see the patients well clothed, well fed, and well housed; will take care of the furniture, the drainage, the laundry, and the farm; and who will, above all, keep down expenses. These are the qualities which the young asylum medical officer sees valued by his committee. These are the qualities which he sees considered in awarding promotion; these, in short, are the qualities that pay; and these are the qualities that he cultivates. No one connected with the asylum cares whether he is or is not efficient as a medical man. Probably there is no one connected with the asylum who is capable of judging whether he is or is not efficient in this capacity. But everyone can tell whether he is or is not a good administrator, and as this is the only quality that is valued, so this is the only quality that is cultivated. Nothing is easier, nothing more obvious, than to sneer at the asylum doctor for his devotion to the farm and the laundry, the drainage and the maintenance rate, to the neglect of his medical functions; but those who thus disparage him should remember that his first duty is to his employers and paymasters, and that he gives them, and is bound to give them, the kind of services which is expected of him and for which he is paid. There are not hours enough in the day, nor is there sufficient power of work in human nature, to enable a man to administer a large institution and at the same time keep himself abreast of the knowledge of the day in a science that advances at the rate medical science does. Still less do the conditions of time and work suffer him to make contributions to that advance. But, it may be said, the junior staff, who are not much burdened with administrative duties, surely they have time and opportunity for scientific work? No doubt they have, but it is scarcely to be expected that their time will be so expended. First there is the lack of incentive; such work does not pay. It does nothing to advance their future prospects. It is no help to promotion. Again, there is the loss of habit, and the absence of that intellectual atmosphere which is such a powerful aid to research in hospitals with medical schools attached. It needs a Darwin to maintain an enthusiasm for science in such circumstances; and it is no reproach to an average medical man that he is not a Darwin.

No; the conditions necessary for scientific research into insanity do not exist and cannot be provided in public rate-supported asylums. The number of beds allotted to each medical officer in an asylum is about four hundred, and he has no assistant of any kind except the nurses. The work cannot be undertaken in public asylums without an expenditure which the ratepayer would not sanction and ought not to be asked to sanction.

